# Reinterpretation on a comparison of cytological adequacy between 23- and 25-gauge in thyroidology: smaller needle gauges "ratio"nale or (over)use it?

**DOI:** 10.1590/1806-9282.20240874

**Published:** 2024-12-02

**Authors:** Demet Sengul, Ilker Sengul

**Affiliations:** 1 Giresun University, Faculty of Medicine, Department of Pathology – Giresun, Turkey.; 2 Giresun University, Faculty of Medicine, Division of Endocrine Surgery – Giresun, Turkey.; 3 Giresun University, Faculty of Medicine, Department of General Surgery – Giresun, Turkey.

Dear Editor,

A Deucalione, the delicate and vital endocrine gland, the thyroid, disorders and therapeutic options remain their significance in human beings^1-3^. We read with great interest and respect to the research article entitled: "Comparison of Cytological Adequacy between 23- and 25-Gauge (G) in Ultrasound-Guided Fine-Needle Aspiration of Thyroid Nodules: A Single-Center Prospective Study." This beneficial research seems to demand a determining comparison of the diagnostic cytology quality of (US)-guided fine-needle aspiration (FNA) in thyroid nodules with the 23- and 25-gauge (G) needles in a total of 177 nodules in 158 outpatients for 18 months^4^. Puga et al.^4^ declared in their study that the 23-G needle possessed higher cytologic adequacy than the thinner one. The authors emphasized, in particular, that the needle gauge is an independent predictor of The Bethesda System for Reporting Thyroid Cytopathology (TBSRTC), non-diagnostic versus 25-G, simplifying an accurate diagnosis for thyroid nodules, although they emphasized no uniform standards for which type of needle to use have been announced to date. In addition, they have mentioned Category I and III as the indeterminate results throughout their aforementioned enrolment of the favorable article. However, a wide range of 20–27-G in size needles have been used for FNA applications in different geographic regions worldwide, that is, 25–27-G in most Western countries and 21–22-G in Japan^5^. As such, some authors alleged the non-diagnostic rates of 22- and 25-G needles were 18.5 and 21.0%, respectively^6^. However, many authors have demonstrated no significant difference in the adequacy rates of the cytologic samples of the thyroid gland achieved with finer and thicker needles^7^. We reported a retrospective study, a sum of 500 nodules in 425 eligible consecutive outpatients for 38 months, involving US-guided FNA (US-FNA) with a surgeon-performed US in thyroid nodules with 27-G fine needles with a reasonably low rate, 9.0%, of Category I, TBSRTC^8^. Additionally, although Category I, TBSRTC, has been defined as non-diagnostic, indeterminate cytology has been terminated as Category III, IV, and V by the American Thyroid Association Management Guidelines for Adult Patients with Thyroid Nodules and Differentiated Thyroid Cancer and most authorities in thyroidology, globally^9^. Moreover, which edition of TBSRTC was utilized by decision-making providers to achieve accurate cytologic diagnosis for those suspicious thyroid nodules: (i) first, (ii) second, or (iii) third (the current one in 2023), by including or excluding the non-invasive follicular thyroid neoplasm with papillarylike nuclear features (NIFTP)^10-12^? Would these conditions, edition, and embracement of NIFTP alter the outcomes of this study? Furthermore, apparently, the criteria used for the decision to perform the FNA procedure for the nodules have not been notified.

As a consequence, the debate is still ongoing on the accuracy of needle size, per se, involving a broad range from 21- to 27-G, globally, for thyroid health providers and Thyroidologists. Of note, some authors have declared that not much finer needles lead to less non-diagnostic cytologic results. However, we might suggest utilizing a 27-G fine needle in the practice of US-FNA procedure for suspicious thyroid nodules with reasonably low rates of non-diagnostic cytology and probably with low severity of pain, which has recently been published in the 67th and 68th volumes of Rev Assoc Med Bras^13-23^. We believe a full appreciation of needle size is dependent on a thorough understanding of its accuracy in the regulation of optimal cytologic diagnoses in Thyroidology. As a matter of fact, this issue merits further investigation. We thank Puga et al.^4^ for their valuable study.
